# Carbon efficient production of chemicals with yeasts

**DOI:** 10.1002/yea.3909

**Published:** 2023-11-23

**Authors:** Evelyn Vásquez Castro, Golnaz Memari, Özge Ata, Diethard Mattanovich

**Affiliations:** ^1^ Austrian Centre of Industrial Biotechnology (ACIB) Vienna Austria; ^2^ University of Natural Resources and Life Sciences, Department of Biotechnology Institute of Microbiology and Microbial Biotechnology Vienna Austria

**Keywords:** carbon balance, bioeconomy, industrial biotechnology, metabolic engineering

## Abstract

Microbial metabolism offers a wide variety of opportunities to produce chemicals from renewable resources. Employing such processes of industrial biotechnology provides valuable means to fight climate change by replacing fossil feedstocks by renewable substrate to reduce or even revert carbon emission. Several yeast species are well suited chassis organisms for this purpose, illustrated by the fact that the still largest microbial production of a chemical, namely bioethanol is based on yeast. Although production of ethanol and some other chemicals is highly efficient, this is not the case for many desired bulk chemicals. One reason for low efficiency is carbon loss, which decreases the product yield and increases the share of total production costs that is taken by substrate costs. Here we discuss the causes for carbon loss in metabolic processes, approaches to avoid carbon loss, as well as opportunities to incorporate carbon from CO_2_, based on the electron balance of pathways. These aspects of carbon efficiency are illustrated for the production of succinic acid from a diversity of substrates using different pathways.

## Graphical abstract

Production of chemicals with yeasts may be hampered by low yield due to carbon loss. The causes for carbon loss in metabolic processes are discussed, as well as approaches to avoid carbon loss and opportunities to incorporate carbon from CO_2_.

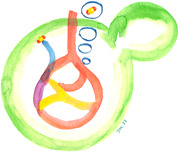

## 1 INTRODUCTION

The rapid increase of atmospheric carbon dioxide concentration as a major cause for global warming is a clear indication that we need to base our economy on renewable, in best case bio‐based feedstocks rather than further exploiting fossil resources. Biotechnology offers excellent opportunities to produce many goods of our daily use, or their (bio)chemical precursors, from biogenic raw materials. Among potential production organisms mainly bacteria and yeasts are being discussed as chassis cells for future synthetic biology applications. Yeasts can utilize different feedstocks from a variety of sources: so‐called first‐generation feedstocks are derived directly from agricultural production (mainly sugar and starch). Second‐generation feedstocks are byproducts from processing of agricultural raw materials, such as lignocellulosic sugars derived from straw or corn stover, or glycerol from biodiesel production. These substrates still base on agricultural production and compete with production of food, animal feed, and plant fiber materials. Recently, the interest in single‐carbon feedstocks has re‐gained momentum—a field where the ability of methylotrophic yeasts to utilize methanol plays a key role.

Carbon efficiency is one of the main factors defining the feasibility of a process, being the main deciding factor for yield of product per substrate. Two factors determine carbon efficiency: (1) the electron balance of the process from substrate to product, easily calculated by the degrees of reduction of substrate and product; (2) the fact that nature has evolved existing metabolic pathways mainly for fast rates rather than for highest carbon yield. The first factor is intrinsically linked to the chemical composition of substrate and product. The second, however, can potentially be overcome by redesigning metabolic pathways so that carbon is conserved or, at best, even assimilated during product formation. Understanding these determinants allows us to understand the limitations of natural metabolic processes, the biochemical limits and the opportunities to extend the synthetic processes beyond the limits of Nature's “toolbox.”

In this review, we discuss the principles of metabolic redox balancing, illustrated with the main substrates and primary metabolite products in yeast biotechnology, and we will outline strategies to design carbon‐saving metabolic routes towards high carbon yield in the bio‐production of chemicals with yeasts.

## 2 REDOX BALANCE IN YEAST METABOLISM

The metabolic pathway efficiency for successful bioproduction of chemical compounds depends on different features such as redox balance, energy balance, thermodynamic feasibility, stoichiometric balance, flux coupling, feedback repression, product toxicity, kinetics, to name the most important (Porro et al., 2014). The cell metabolism always needs to be redox balanced by the transfer all the electrons from the substrate to the different metabolites to sustain cellular growth and maintenance. Therefore, the design of an optimal biosynthetic pathway for production of a desired metabolite should be redox‐neutral and should reach a pathway yield (*Y* ^P^) equal or very close to the maximum theoretical yield (*Y* ^E^) of the substrate–target product combination (Folch et al., 2021). *Y* ^P^ depends on the pathway involved and is determined based on its stoichiometry, whereas *Y* ^E^ is the maximum amount of product that can be formed from the substrate and is calculated from the ratio *γ*_S_/*γ*_P_, where *γ*_S_ and *γ*_P_ are the degrees of reduction of substrate and product, respectively (Dugar & Stephanopoulos, 2011; Vuoristo et al., 2016). The degree of reduction may be defined as the number of equivalents of the available electrons per carbon atom of the compound (Shuler & Kargi, 2002). Consequently, *Y* ^E^ considers the electron balance of the conversion of substrate to product which may require carbon loss due to decarboxylation or enable additional carbon uptake by carboxylation, respectively.

**Figure 1:**
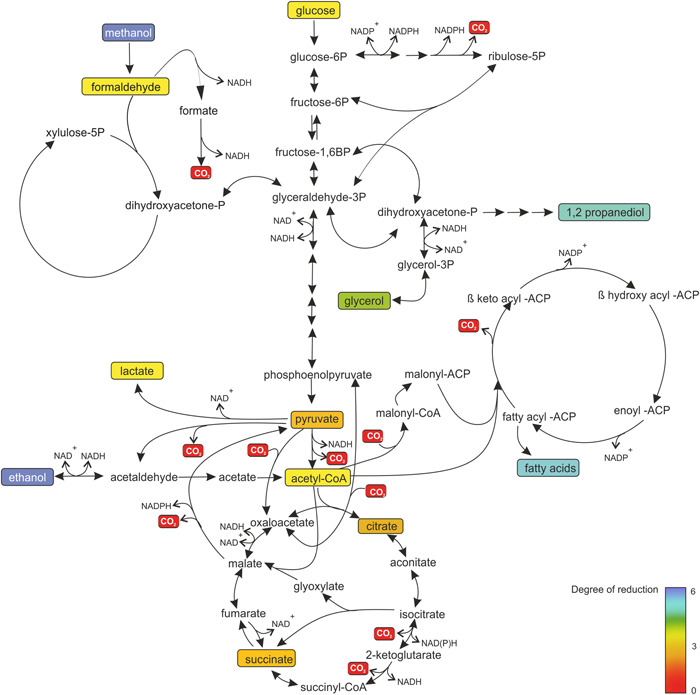
Pathways involved in the central carbon metabolism of yeasts, highlighting the relation between carboxylation/decarboxylation steps and the change in the degree of reduction of substrates and products. Degree of reduction of the respective substrates, intermediates, and products is indicated by a color code ranging from red (*γ* = 0) over yellow (*γ* = 4) to blue (*γ* = 6).

Figure 1 depicts the pathways structure involved in the central metabolism in yeast. Glucose, glycerol, and methanol are shown as representative carbon feedstocks and some products synthesized in yeast are highlighted with an emphasis on redox steps and carboxylations or decarboxylations, respectively.

Depending on the degree of reduction of substrate and target metabolite, three possible scenarios can be described: First, when the substrate and target metabolite have the same degree of reduction, it can lead to an ideal full conversion of substrate into the product. Even though the practical product yields could approach *Y* ^E^, native metabolic processes generate by‐products for biomass formation and cell maintenance, which inevitably reduce the product yield. Lactic acid (*γ* = 4.0) is an example of this possible metabolic scenario since its production conserves the degree of reduction of glucose (*γ* = 4.0). Therefore, the redox‐neutral pathway for lactic acid production, which is also stoichiometrically balanced, can generate ATP, thus allowing to achieve near maximum yields. In general, it is not that common to find such pathways, which produce no excess reducing power, for other substrate–product pairs (Folch et al., 2021).

Second, when the product is more reduced than the substrate, additional oxidizing equivalents (NAD^+^, NADP^+^, FADH^+^) are generated by the reducing reactions required for product formation. To reduce those oxidizing equivalents again, the cell simultaneously needs to oxidize carbon to carbon dioxide (CO_2_) and/or other byproducts (either in the same metabolic pathway or in others such as the pentose phosphate pathway [PPP], the tricarboxylic acid [TCA] cycle, or the xylulose monophosphate [XuMP] cycle) to maintain the redox balance. This complete process may compromise the overall efficiency of the substrate conversion towards the target metabolite. Metabolites such as fatty acids, ethanol and glycerol are more reduced than glucose.

Fatty acid (e.g., *γ*_palmitic acid_ = 5.75) formation from glucose releases CO_2_ in the chain elongation cycle, due to the high NADPH demand, resulting in a substrate loss that decreases the fatty acid yield (Hu et al., 2019; Sheng & Feng, 2015). Yu et al. (2022) succeeded in achieving 40% of theoretical yield for fatty acid production in *Saccharomyces cerevisiae* by implementing a synthetic reductive metabolic pathway, characterized by a repeated decarboxylation cycle, which can supply extra NADH, NADPH, and ATP to the cell metabolism. This approach rewired the energy metabolism towards improving the yield of highly reduced metabolites (Yu et al., 2022).

Ethanol production from glucose also oxidizes part of the substrate to CO_2_ and glycerol as byproducts due to the required input of NADH. However, the native yeast pathway for ethanol fermentation conserves the degree of reduction of glucose (*γ* = 4.0), as CO_2_ and ethanol, together as final products, have an overall average reduction degree of *γ* = 4.0. Thus, the metabolic pathway is extremely efficient from a yield perspective, losing only 4%–5% of carbon source into glycerol formation (Nissen et al., 2000). Similarly, to produce 1,2‐propanediol (1,2‐PDO) (*γ* = 5.33) using glycerol (*γ* = 4.66) as the sole carbon source, Islam et al. (2017) employed genetic modifications in *S. cerevisiae* to supply extra NADH for boosting 1,2‐PDO synthesis, reaching the highest titer >4 g/L 1,2‐PDO, in yeast thus far.

Third, when the product is less reduced than the substrate, the metabolism generates reducing equivalents along with the product. A common mechanism to reoxidize the excess reducing equivalents is their oxidation via the respiratory chain, leading to an ATP surplus, and/or the release of metabolic heat. Hereby, the product yield is lower than the theoretical maximum that could be achieved based on the available electrons. Alternatively, excess reducing equivalents can be consumed by reduction of a fraction of the carbon source to a reduced by‐product. This substrate‐product combination has the potential to incorporate carbon to improve the target metabolite yield. For instance, in the production of citric acid (*γ* = 3.0) from glucose, there is an energy overflow due to NADH formation, which implies that cells can gain energy simply by making the target compound—at the expense of yield loss. Thus, the native biochemical route to synthesize citric acid works under suboptimal efficiency, representing an opportunity to achieve near‐maximum theoretical yields by incorporation of carbon.

Therefore, the substrate selection for a desired product could be based on γ_S_ and *γ*_P_ to maximize the yield. Thus, glucose which is the preferred carbon source for yeast can be used to synthesize products like ethanol (plus CO_2_) or lactic acid, which have the same *γ* as glucose. Even though glucose is the favored substrate, this carbon source competes directly with food and feed production and the cost of glucose is rising in recent years due to significant increase of biotechnological processes (Steiger et al., 2017). Therefore, several cheaper carbon sources including glycerol, methanol and CO_2_ are considered as promising substrates (An et al., 2021).

Glycerol results as a byproduct from biodiesel production. This carbon source has a higher *γ* compared with sugars, which makes it an interesting alternative substrate for the production of three‐carbon reduced target molecules such as 1,2‐PDO.

Methanol (*γ* = 6.0) is a highly reduced one‐carbon (C1) feedstock that can be obtained in an environment‐friendly manner. A major advantage of using methanol as carbon source is its reducing power that forms NADH and subsequently ATP in microorganisms like methylotrophic yeasts. Yeasts, however, lose one NADH per methanol due to the first pathway reaction, oxidizing methanol to formaldehyde with oxygen as the electron acceptor. It was shown recently that *Komagataella phaffii* (*Pichia pastoris*) is able to utilize methanol in a more efficient way by overexpressing a native alcohol dehydrogenase (Adh2) in alcohol oxidase deficient strains (Mut^−^), which leads to additional NADH and ATP yield per methanol. Consequently, Mut^−^ Adh2 overexpressing strains increased the productivity of a heterologous protein at low oxygen uptake and heat dissipation (Zavec et al., 2021). This approach emphasizes the potential of methanol as an emerging biotechnological substrate for yeast‐based processes.

Another promising carbon feedstock is CO_2_, which acts as a greenhouse gas in the atmosphere heating our planet. CO_2_ is a highly oxidized compound (*γ* = 0) that can be reduced to be incorporated into organic compounds for biosynthesis by autotrophic organisms such as plants and cyanobacteria. Therefore, an interesting approach to channel CO_2_ into the metabolism of yeast is the mixed‐substrate conversion, where CO_2_ along with another carbon source could be converted into products with lower reduction degree than the co‐substrate. For instance, in the biosynthesis of organic acids, which have lower *γ* than glucose, such as citric acid, itaconic acid, and succinic acid (SA), this strategy could be used to incorporate CO_2_ in an industrial process to improve the carbon yield (An et al., 2021; Steiger et al., 2017).

## 3 HOW CAN WE BALANCE THE DEGREE OF REDUCTION OF THE PRODUCTS?

Microbial metabolic processes have evolved by selection for fast cell growth rather than the production of a specific product (Yu et al., 2022). Consequently, fast turnover rates are optimized rather than high carbon yield. Therefore, the capability of the cells to improve carbon conservation during their metabolism is one of the greatest metabolic engineering challenges that has hindered achieving high yields of valuable chemicals in microbial factories.

In previous reviews, the seven natural carbon fixation pathways through which CO_2_ can enter the metabolism of autotrophs, the synthetic CO_2_ assimilation pathways, as well as exploiting synthetic biology tools to rewire the carbon metabolism of heterotrophs to optimize carbon conservation have been discussed extensively (Corea et al., 2023; François et al., 2020; Kim et al., 2022). The current mini‐review focusses on the metabolic engineering of yeast to maximize carbon conservation with implemented strategies for the incorporation of CO_2_ fixation steps along with strategies to avoid unnecessary decarboxylation steps in the cell.

### 3.1 Incorporate CO_2_ as a substrate

Carbon fixation is the biochemical process allowing to turn inorganic carbon into organic compounds, thus providing the backbone of the cellular building blocks. There are different routes for inorganic carbon integration into the metabolism of the cell: Carboxylation reactions where a CO_2_ molecule is incorporated into an organic compound and CO_2_ reduction reactions, where CO_2_ is converted to formate or carbon monoxide, which can be later assimilated into biomass (Cotton et al., 2018).

Carboxylation reactions are catalyzed by carboxylases. Some of these enzymes can be involved either in autotrophic CO_2_ fixation pathways, for example, ribulose 1,5‐bisphosphate carboxylase (RuBisCO) involved in the Calvin–Benson–Bassham (CBB) cycle) or in natural microbial pathways which provide central precursors to the cell (e.g., phosphoenolpyruvate carboxylase and pyruvate carboxylase involved in the glycolytic oxaloacetate pathway (Erb, 2011). On the other hand, examples of carbon reduction are the reactions catalyzed by formate dehydrogenase or CO dehydrogenase that are involved in the Wood–Ljungdahl pathway (reductive acetyl‐coenzyme A [acetyl‐CoA] pathway), where CO_2_ reduction to formate or carbon monoxide delivers carbon to be used for acetyl‐CoA formation (Cotton et al., 2018; Erb, 2011).

#### 3.1.1 Expression of heterologous CBB enzymes for CO_2_ fixation in yeasts

In *S. cerevisiae*‐based bioethanol production, the formation of glycerol hampers the cost‐effective production of ethanol from sugars. Rewiring the cellular metabolism of this yeast to use CO_2_ as an electron acceptor is an attractive strategy implemented by Guadalupe‐Medina et al. (2013). In this study, functional expression of the CBB cycle enzymes phosphoribulokinase (PRK) and RuBisCO resulted in the conversion of CO_2_ as a major product of alcoholic fermentation to ribulose 5‐phosphate, a typical intermediate of the PPP pathway. Incorporation of CO_2_ in the central carbon metabolism of the yeast and the creation of another metabolic pathway for the production of ribulose 5‐phosphate yielded in 90% reduction of glycerol byproduct and 10% increase in ethanol production in chemostat from glucose and galactose.

In another study done by Xia et al. (2017), they used a partial CBB cycle in a xylose utilizing *S. cerevisiae* strain to increase bioethanol production yield and to lower the yields of xylitol and glycerol as byproducts indicating the redox imbalance during anaerobic fermentation of xylose. Re‐assimilation of the CO_2_ generated after decarboxylation of pyruvate was achieved by overexpressing RuBisCO from *Rhodospirillum rubrum* and PRK from *Spinacia oleracea*. The resulting strain has the advantages of lignocellulosic ethanol production and CO_2_ conservation (Xia et al., 2017).

The full implementation of the CBB cycle in yeast has been achieved by Gassler et al. who converted the XuMP cycle of methylotrophic *K. phaffii* into the CO_2_ assimilating CBB cycle, creating an organo‐autotrophic yeast (Gassler et al., 2020). By adding reactions towards organic acids the production of lactic and itaconic acid from CO_2_ as only carbon source was demonstrated (Baumschabl et al., 2022). In the resulting organo‐autotrophic strain, energy and reducing power required for growth are supplemented by methanol oxidation via the dissimilatory pathway to CO_2_, so that the net CO_2_ balance is reduced by the energy demand. Designing pathways to incorporate the carbon from methanol along with CO_2_ into biomass can be a way to improve the CO_2_ balance markedly, as the reduced carbon of methanol is directly incorporated into products together with CO_2_ instead of serving only as the electron and energy donor for a rather energy intensive pathway to reduce the carbon of CO_2_.

#### 3.1.2 Reductive glycine pathway

The reductive glycine pathway is considered as the most energy‐efficient pathway for aerobic growth on formate (Bar‐Even et al., 2013). CO_2_ is co‐assimilated with a methylene group from formate by the reverse reaction of the glycine cleavage system. With a further methylene group serine is formed, entering the central carbon metabolism. Although all enzymes of the reductive glycine pathway exist natively in *S. cerevisiae*, cells are not adapted to grow on formate as it is not a common substrate in the relevant natural environments. Overexpression of only endogenous enzymes in the yeast *S. cerevisiae* resulted in an activated reductive glycine pathway that enabled glycine biosynthesis from formate and CO_2_ and maintained growth of a glycine auxotrophic strain without the addition of glycine. This growth relies on a high concentration of CO_2_ (10%), which is needed to support the pathway both thermodynamically and kinetically (de la Cruz et al., 2019). Furthermore, a native oxygen tolerant reductive glycine pathway has been recently discovered in the yeast *K. phaffii*. However, the activity of this pathway for production of glycine is not high enough to support growth without further engineering of cell metabolism (Mitic et al., 2022).

#### 3.1.3 Reductive branch of the TCA cycle (rTCA)

The reductive TCA cycle (rTCA) is a cyclic pathway of CO_2_ fixation found in prokaryotes. rTCA is a reversal of the widespread TCA cycle and forms one molecule of acetyl‐CoA by fixing two CO_2_ molecules (Correa et al., 2023). Most reactions of the TCA cycle are reversible with the present enzymes. In addition three enzymes are necessary to catalyze reverse reactions: ATP citrate lyase, fumarate reductase and 2‐ketoglutarate:ferredoxin oxidoreductase (Erb, 2011; Kim & Gadd, 2019). Recently, it was also found that a reversible TCA cycle is present in two anaerobic bacteria without needing an ATP citrate lyase. Instead, these microorganisms possess a reversible citrate synthase that requires reduced ferredoxin, which enables the TCA cycle to run in reverse (Mall et al., 2018; Nunoura et al., 2018).

The full rTCA cycle has, however, not been realized in yeasts up to now. A partial rTCA cycle has been implemented in *S. cerevisiae* to produce succinic and malic acid. Yan et al. (2014) engineered a pdc and fum1‐deficient strain that overexpressed genes encoding pyruvate carboxylase (*PYC2*) and the first three enzymes of the rTCA cycle (*MDH3R*, *Escherichia coli* FumC and *FRDS1*) to assemble the pathway from oxaloacetate to succinate. They could produce up to 13 g/L succinate with a yield of 0.21 mol/mol glucose after implementing also some bioprocess engineering strategies (Yan et al., 2014). Kang and co‐workers reported the production of 61.2 g/L of malic acid from xylose in a *S. cerevisiae* engineered strain harboring enzymes that are part of the rTCA pathway (Kang et al., 2022). In a more recent paper, Malubhoy et al. (2022) also achieved up to 35 g/L succinate and the highest yield of 0.63 mol/mol glycerol via the rTCA pathway along with a net CO_2_ fixation (Malubhoy et al., 2022).

### 3.2 Avoiding unnecessary decarboxylation

Biological decarboxylation is a reaction mechanism that releases CO_2_ mostly from carboxylic acids. Decarboxylations occur mainly in catabolic pathways ‐ glycolysis, the PPP and the TCA cycle—and they are often connected with oxidations and so they regenerate reduced cofactors like NADH and NADPH. Due to the interconnections of catabolic and anabolic pathways in the central carbon metabolism, decarboxylations occur also on the route to precursor metabolites for the final desired products. Any decarboxylation reaction in a pathway decreases the carbon yield from substrate to product and should be avoided if possible. Thus, under unnecessary decarboxylation, we understand a decarboxylation step that can be bypassed for the synthesis of a desired metabolite, to improve the carbon conservation in the cell.

For instance, acetyl‐CoA is an important two‐carbon metabolite produced by the decarboxylation of pyruvate, which leads to 33% loss of carbon as CO_2_, decreasing the theoretical product yield of any process involving acetyl‐CoA (François et al., 2020). Acetyl‐CoA is a substrate in different biological processes such as the TCA cycle, fatty acid biosynthesis, and amino acid biosynthesis. Acetyl‐CoA is also a metabolic intermediate for many industrially relevant products such as lipids, isoprenoids, 3‐hydroxypropionate, citric acid, amino acids, and many more (Ku et al., 2020).

Therefore, to overcome the carbon loss in acetyl‐CoA synthesis, significant efforts have been dedicated to design new carbon conservation pathways that avoid the unnecessary decarboxylation step. Hellgren et al. (2020) expanded the linear phosphoketolase pathway to a new design of the nonoxidative glycolysis cycle to create a circular carbon‐conserving pathway (glycolysis alternative high carbon yield cycle, GATHCYC) in *S. cerevisiae* this pathway can produce three acetyl‐CoA from one fructose 6‐phosphate (F6P), without carbon loss in the form of CO_2_. The authors used a phosphoketolase enzyme that can irreversibly split different sugar phosphates to acetyl phosphate (AcP) and another sugar phosphate. Namely, F6P, xylulose 5‐phosphate or sedoheptulose 7‐phosphate are split into AcP and erythrose 4‐phosphate, glyceraldehyde 3‐phosphate or ribose 5‐phosphate, respectively. Then AcP is coupled with a phosphotransacetylase for the production of acetyl‐CoA as an alternative route to bypass pyruvate dehydrogenase (PDH) for creating cytosolic acetyl‐CoA without carbon loss. The expression of GATHCYC as carbon‐conserving route increased 3‐hydroxypropionic acid titers by 109% (from about 1 to 2 g/L) at the end of the glucose phase. Zhou et al. (2023) also introduced the GATHCYC pathway along with other genetic engineering modifications into a n‐butanol producing *S. cerevisiae* strain that showed an increase in the acetyl‐CoA supply that improved the n‐butanol titer up to 1.75 g/L with a decrease of 35.2% of the total CO_2_ (Zhou et al., 2023).

## 4 SUCCINIC ACID PRODUCTION AS A CASE STUDY

In addition to redox balance and carbon conservation, thermodynamic feasibility and energy balance are also key factors in designing optimal metabolic pathway configurations. The thermodynamic feasibility is provided by the Gibbs free energy change under physiologically relevant standard conditions (Δ_r_ *G*’^m^), which determines whether the metabolic pathway is possible. The cellular energy should be also balanced to produce more of a target compound, since products requiring metabolic energy lead to a substrate loss to meet the energy demand, while the oxidized products lead to energy surplus that could produce heat dissipation (requiring intense cooling systems during fermentation) (Porro et al., 2014).

**Figure 2:**
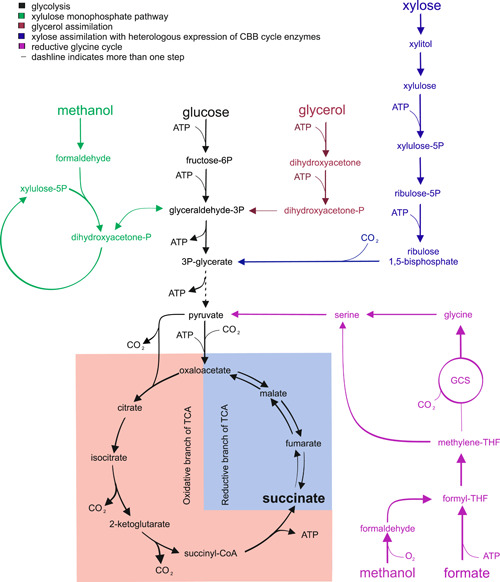
Production of succinate with incorporation of CO_2_ as cosubstrate from the oxidative or reductive branches of the tricarboxylic acid cycle, respectively. For clarity the glyoxylate shunt is not shown in the figure.

SA is an organic acid produced as an intermediate in the TCA cycle. SA has been identified as one of the top value platform chemicals that can be obtained from carbohydrate biomass (Becker et al., 2015). In fact, SA can be converted into 1,4‐butanediol, butadiene, tetrahydrofuran and bio‐based polymers (Liu et al., 2022). These chemicals need to be cost‐competitive with their conventional petrochemical production (Vuoristo et al., 2016), therefore bringing maximum pathway yield into proximity to theoretical yield of product per substrate is of interest. In this chapter, we are focusing on different approaches to produce SA (Figure 2). In particular, we evaluate ATP stoichiometry, redox‐balance, CO_2_ fixation, thermodynamic feasibility and carbon conservation of different native and engineered SA‐forming pathways (see Table 1).

**Table 1:** Comparison of different native and engineered pathways for SA production in yeast.

TCA cycle	Pathway	Stoichiometry	CO_2_ as additional carbon source	ATP/SA (mol/mol)^a^	NADH/SA (mol/mol)^a^, ^b^	CO_2_/SA (mol/mol)^a^	Δ_r_ *G*’^m^ (kJ/mol SA)^c^	*Υ* ^P^ (Cmol SA/Cmol of organic substrate)^d^	*Υ* ^E^ (Cmol SA/Cmol of organic substrate)^e^	Current Y in yeast (Cmol SA/Cmol of organic substrate)	References
Reductive branch of TCA	Glucose assimilation via glycolysis	Glucose + 2 CO_2_ + 2 NADH ⇌ 2 Succinate + 2 NAD + 2 H_2_O	Yes	0	−1	−1	−134.05 ± 6.95	1.33	1.14	0.21	Yan et al. (2014)
	Glycerol assimilation	Glycerol + CO_2_ ⇌ Succinate + H_2_O	Yes	0	0	−1	−110.5 ± 7.9	1.33	1.33	0.63	Malubhoy et al. (2022)
	Xylose assimilation via glycolysis and partial CBB cycle	Xylose + 3 CO_2_ + 2 ATP + 4 NADH ⇌ 2 Succinate + 2 ADP + 2 P_i_ + 4 NAD + H_2_O	Yes	−1	−2	−1.5	−160.0 ± 9.8	1.6	1.14	No data	
	Formate assimilation via serine and reductive glycine pathway	2 Formate + 2 CO_2_ + 3 ATP + 5 NADH ⇌ Succinate + 3 ADP + 3 P_i_ + 5 NAD + + H_2_O	Yes	−3	−5	−2	−148.4 ± 14.1	2	0.57	No data	
	Methanol assimilation via serine and reductive glycine pathway	2 Methanol + O_2_ + 2 CO_2_ + 3 ATP + 3 NADH ⇌ Succinate + 3 ADP + 3 P_i_ + 3 NAD + H_2_O	Yes	−3	−3	−2	−624.4 ± 18.5	2	1.71	No data	
	Methanol assimilation via XuMP pathway	3 Methanol + 1.5 O_2_ + CO_2_ + ADP + P_i_ + NADH ⇌ Succinate + ATP + NAD + 5 H_2_O	Yes	1	−1	−1	−689.8 ± 21.3	1.33	1.71	No data	
Oxidative branch of TCA	Glucose assimilation via glycolysis	Glucose + 2 ADP + 5 NAD + 2 P_i_ ⇌ Succinate + 2 CO_2_ + 2 ATP + 5 NADH	No	2	5	2	−195.3 ± 12.9	0.67	1.14	0.024	Ito et al. (2014)
	Glycerol assimilation	2 Glycerol + 2 ADP + 2 P_i_ + 7 NAD ⇌ Succinate + 2 CO_2_ + 2 ATP + 7 NADH	No	2	7	2	−148.2 ± 15.1	0.67	1.33	0.33	Yuzbashev et al. (2016)
	Xylose assimilation via glycolysis and partial CBB cycle	Xylose + 3 NAD + H_2_O ⇌ Succinate + CO_2_ + 3 NADH	No	0	3	1	−247.1 ± 8.1	0.8	1.14	No data	
	Formate assimilation via serine and reductive glycine pathway	4 Formate + 4 ATP + 3 NADH ⇌ Succinate + 4 ADP + 4 P_i_ + 3 NAD	No	−4	−3	0	−224.0 ± 11.6	1	0.57	No data	
	Methanol assimilation via serine and reductive glycine pathway	4 Methanol + 2 O_2_ + 4 ATP + NAD ⇌ Succinate + 4 ADP + 4 P_i_ + NADH	No	−4	1	0	−1175.9 ± 26.9	1	1.71	No data	
	Methanol assimilation via xylulose monophosphate pathway	6 Methanol + 3 O_2_ + 5 NAD + 4 ADP + 4 P_i_ ⇌ Succinate + 2 CO_2_ + 4 ATP + 5 NADH + 8 H_2_O	No	4	5	2	−1306.7 ± 41.6	0.67	1.71	0.16	Zhang et al. (2023)
Partial TCA and GS	Glucose assimilation via glycolysis and GATHCYC pathway	7 Glucose + 6 CO_2_ + 4 ATP ⇌ 12 Succinate + 4 ADP + 4 P_i_ + 2 H_2_O	Yes	−0.33	0	−0.5	−175.43 ± 4.72	1.14	1.14	No data	

Abbreviations: CBB, Calvin–Benson–Bassham; GATHCYC, glycolysis alternative high carbon yield cycle; GS, glyoxylate shunt; SA, succinic acid; TCA, tricarboxylic acid cycle; XuMP, xylulose monophosphate.

^a^ Negative or positive values mean consumption or production, respectively.

^b^ NADPH was replaced by NADH in the calculations.

^c^ The Gibbs free energy change of reaction (Δ_r_ *G*’^m^) values are calculated with equilibrator (http://equilibrator.weizmann.ac.il) and normalized to the number of moles of SA in the reaction. Δ_r_ *G*’^m^ values are calculated for physiologically meaningful parameters with reactants concentrations of 1 mM, a pressure of 1 bar and a temperature of 298.15 K.

^d^ *Y* ^P^ represents the maximum pathway yield. It depends on the pathway involved and is calculated from pathway stoichiometry without considering the substrate demand for the redox or ATP balance.

^e^ *Υ* ^E^ represents the theoretical maximum yield.

There are three different routes that could be exploited for SA production: (i) the oxidative and (ii) the reductive branch of the TCA cycle (oTCA and rTCA respectively), as well as (iii) the glyoxylate shunt (GS). The oTCA has lower theoretical maximum yield but aerobic SA production has developed attributes of reduced byproduct and a thermodynamically more favorable metabolism (Ito et al., 2014). The GS is an alternative to oTCA for the production of SA and it prevents carbon loss by bypassing two decarboxylation steps between isocitrate and succinyl‐CoA (Raab & Lang, 2011) and provides extra NADH. The reductive branch of TCA cycle, on the other hand, enables CO_2_ fixation and provides almost two‐fold higher *Y* ^P^ compared with the oTCA route. It should be noted, however, that *Y* ^P^ is a local parameter considering only the net stoichiometry of the respective pathway, but not any carbon loss during regeneration of NAD(P)H or ATP. *Y* ^E^, on the other hand, is a global parameter considering the electron balance and therefore also NAD(P)H regeneration. Therefore, *Y* ^P^ can be even higher than *Y* ^E^ in some instances (Table 1). Production of SA through rTCA mainly happens in rumen bacteria under anaerobic conditions (Ito et al., 2014). Generally, rTCA is thermodynamically unfavorable in yeasts and leads to the shortage of NADH supply for the cell. The formation of succinate via the rTCA involves the back reactions starting from oxaloacetate towards succinate, which are catalyzed by malate dehydrogenase, fumarase, and fumarate reductase (Raab & Lang, 2011).

As explained earlier in Section 2, there are several engineering strategies applied in yeasts for the production of value‐added chemicals with incorporation of CO_2_ as substrate. Here we chose SA as the final product to make a comparison between production of SA from different carbon sources via the reductive or oxidative branch of the TCA cycle. As summarized in Table 1, the Gibbs free energy change of the reaction (Δ_r_ *G*’^m^) per mole SA was calculated for glucose assimilation via glycolysis (Yan et al., 2014), glycerol assimilation (Malubhoy et al., 2022; Yuzbashev et al., 2016), xylose assimilation via partial CBB cycle (Xia et al., 2017), formate or methanol assimilation via the reductive glycine pathway (de la Cruz et al., 2019), and methanol assimilation via the xylulose‐monophosphate pathway (Zhang et al., 2023).

SA production has been explored in yeast, as these microorganisms tolerate lower pH values, reducing the production cost of SA, especially during downstream processing (Becker et al., 2015). As described above, there are different metabolic pathways leading to SA production; however, the rTCA branch has drawn increasing attention since it allows CO_2_ fixation instead of release. In fact, commercial production of SA has been established through the rTCA pathway in an *S. cerevisiae* mutant strain by using glucose as a carbon source. This engineered strain, developed by the company Reverdia, produced 43 g/L of SA in aerobic condition (Van De Graaf et al., 2015).

Although SA production from glucose via glycolysis and rTCA allows to incorporate 1 mol CO_2_/mol SA in the process, the pathway is not redox‐balanced, requiring the input of 1 mol NADH per mol SA. An attractive alternative to get a redox‐neutral SA production with the potential to fix 1 mol CO_2_/mol SA via the rTCA pathway is to use the higher reducing power of glycerol as a carbon source (Figure 2 and Table 1). The combination of glycerol + CO_2_ is another example of the first metabolic scenario presented in Section 1, as the overall reduction degree for both carbon sources is *γ* = 3.5, which is the same as of SA. This approach enabling alternative glycerol utilization has been implemented in yeast. For example, Malubhoy et al. (2022) improved the flux from glycerol to SA via the rTCA pathway in an engineered *S. cerevisiae* strain, resulting in an SA yield of 0.6 g/g glycerol (i.e., 47.1% of the theoretical maximum) and CO_2_ fixation after 72 h of shake flasks cultivation.

**Figure 3:**
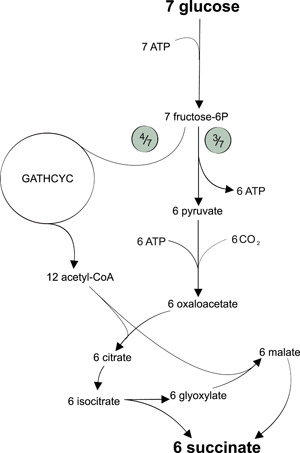
Redox neutral succinate production from a combination of the glycolysis alternative high carbon yield cycle (GATHCYC) along with partial tricarboxylic acid cycle and glyoxylate shunt pathways.

Another interesting approach to achieve redox‐neutral SA production is to use glucose and CO_2_ as co‐substrates. For example, the simultaneous use of glycolysis, GATHCYC and parts of the TCA cycle (Figure 3 and Table 1) could theoretically lead to a closed redox balance with the potential to fix 0.5 mol of CO_2_ for each mol of SA produced. However, this will be at the expense of hydrolysis of 0.33 mol ATP per mol SA which needs to be regenerated, for example, by respiratory consumption of a fraction of glucose. As a consequence, this and other pathways consuming ATP and/or NADH (mainly those following the reductive branch of the TCA cycle) cannot be operated anaerobically but need at least microaerobic conditions which adds another cost factor to the process.

## 5 CONCLUDING REMARKS AND FUTURE PERSPECTIVES

In industrial biotechnology, carbon efficiency of the metabolic pathway determines the impact of substrate costs on the overall product cost structure and the CO_2_ balance of production. It is a major decisive factor whether a specific production is a CO_2_ sink, or is net releasing CO_2_. Carbon is one of the most precious resources for anthropogenic material production, and it is currently treated extremely wastefully, and damaging for the environment. Microbial metabolism enables the use of carbon resources in an environmentally friendly way. Although carbon yield is the key parameter defining the cost structure of raw materials of a process, productivity is of similar importance as it defines the required sizes of production facilities for a given output, and thus the capital expenditures.

The electron balance between substrate(s) and product(s) is a key factor determining theoretical carbon efficiency. Consequently, the choice of a pathway and of substrate (or cosubstrates) determine if the full potential of theoretical yield can be realized. Co‐utilization of CO_2_ with reduced carbon sources is a way to approach carbon efficiency. Among the co‐substrates, single carbon substrates are major resources of the future as they do not consume agricultural products that are better used for human nutrition. Methylotrophic yeasts offer excellent opportunities towards a C1 bioeconomy.

## AUTHOR CONTRIBUTIONS

All authors contributed equally to this work.

## CONFLICT OF INTEREST STATEMENT

The authors declare no conflict of interest.

## Data Availability

Data sharing not applicable to this article, as no data sets were generated or analyzed during the current study.
